# Ins and outs of IRES elements: function and significance

**DOI:** 10.1042/BST20253097

**Published:** 2025-10-28

**Authors:** Encarnacion Martínez-Salas

**Affiliations:** Centro de Biología Molecular Severo Ochoa, CSIC-UAM, Nicolás Cabrera 1, Madrid , 28049, Spain

**Keywords:** bioinformatics, IRES, RNA, RNA-binding proteins, translation initiation

## Abstract

RNA and proteins are key components of all organisms. Internal ribosome entry site (IRES) elements are a diverse type of RNA regulatory structural elements that mediate end-independent, internal translation initiation in viral mRNAs and certain cellular mRNAs translated under stress conditions. Notably, viral IRES elements regulate translation initiation via a dynamic, modular RNA structure organization, which serves as the anchoring site for the ribosome guided by RNA–RNA and/or RNA–protein interactions. The implementation of advanced transcriptomics, proteomics, and computational methodologies has facilitated the identification of novel RNAs potentially translated using cap-independent mechanisms, harboring RNA structural elements with distinctive features. Here, we present a summary of the current understanding of IRES elements, focusing on the molecular functions and the RNA-binding proteins regulating IRES activity.

## Introduction

**Figure 1:**
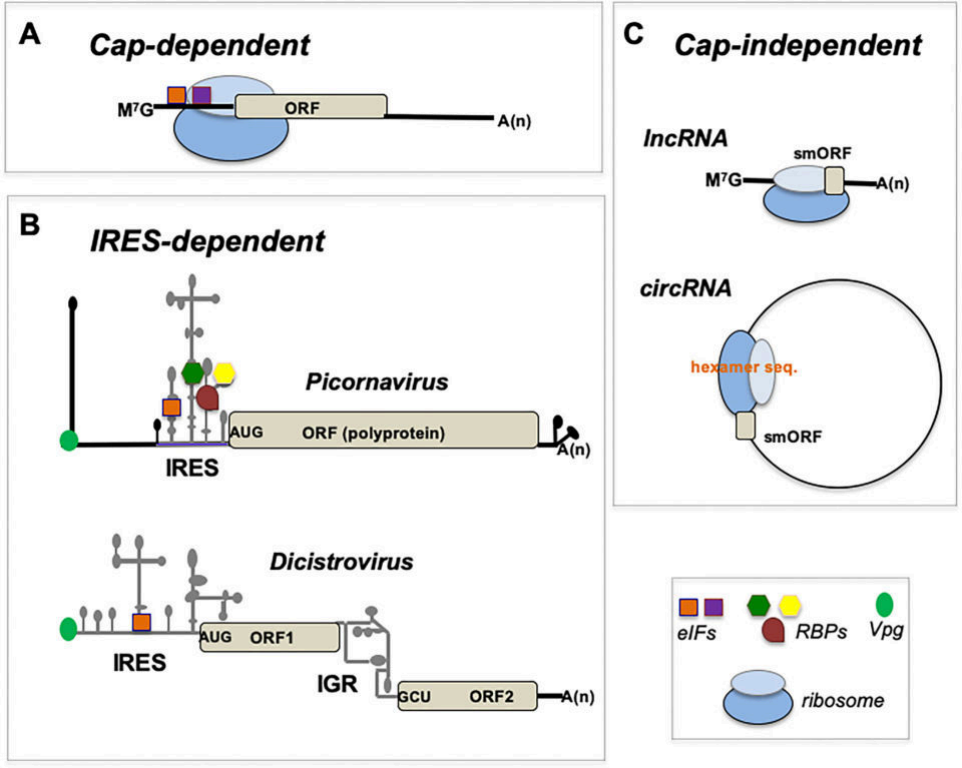
Diagram of translation initiation mechanisms operating in eukaryotic organisms. (**A**) Cap-dependent mechanism operates on the vast majority of capped (M^7^GTP), polyadenylated (A_n_) mRNAs, with relatively short open reading frames (ORFs); eIFs (colored squares) assist the recruitment of ribosomal subunits (light and dark blue circles). (**B**) Viral IRES-dependent translation initiation occurs in uncapped mRNAs with long, heavily structured IRES elements, depicted for picornavirus RNAs (type II) and dicistrovirus RNAs, showing the structured 5′ IRES that initiates translation of ORF1, and the pseudoknotted intergenic region (IGR) that promotes initiation of ORF2 at non-AUG codons (e.g. GCU, GCA, GUU, and CCU) without the assistance of eIFs. A green circle depicts the viral genome protein covalently linked at the 5′ end. (**C**) Cap-independent mechanisms operate in long non-coding RNAs (lncRNAs) and internal initiation in circular RNAs (circRNAs), resulting in the translation of short small open reading frames (smORFs), likely producing microproteins. The three initiation mechanisms shared basic components of the translation machinery (ribosomal subunits, eIFs, and several RBPs). eIFs, eukaryotic initiation factors; IRES, internal ribosome entry site elements; RBPs, RNA-binding proteins.

Regulation of RNA translation accounts for balanced synthesis of all cellular proteins, the major components of most cellular machineries. The process of mRNA translation encompasses a series of regulated steps, from ribosome recycling and translation initiation to elongation and termination. Given that the vast majority of eukaryotic mRNAs harbor an Methyl-7-GTP (M^7^GTP) (cap) residue at the 5′ end, mRNA translation is usually initiated by a cap-dependent mechanism (Figure 1A). Thus, cap-dependent initiation of translation has been considered a dogma in molecular biology [1]. During cap-dependent initiation, the M^7^GTP structure present in most mRNAs allows binding of a complex consisting of three eukaryotic initiation factors (eIFs), the cap-binding protein eIF4E, the RNA helicase eIF4A, and the scaffolding protein eIF4G. Recognition of the 5′ cap by eIF4E allows recruitment of the 43S complex assisted by the multisubunit factor eIF3, including the ternary complex (initiator methionyl-tRNA, eIF2, and GTP), eIF1, eIF1A, eIF5, and the 40S ribosomal subunit. The interaction of eIF4G with the poly(A)-binding protein (PABP), bound to the poly(A)-tail of mRNAs, circularizes the 3′ and 5′ ends of the mRNA, stimulating translation initiation. Under normal conditions, the 43S complex scans the 5′ UTR of the mRNA in a 5′ to 3′ direction in an ATP-dependent manner until an initiator start codon (AUG) is located in optimum sequence context (A/GXXAUGG, where X is any nucleotide). AUG codon recognition triggers joining of the 60S ribosomal subunit and release of eIFs, leading to the assembly of a translation elongation-competent 80S ribosome [2].

The discovery of internal ribosome entry site (IRES) elements in the RNA genome of picornaviruses provided a theoretical framework of translation initiation independent of the 5′ end of the mRNA [3], challenging the paradigm of canonical cap-dependent translation. Since the initial IRES discovery in picornavirus RNAs, genetic mechanisms of internal translation initiation have been systematically studied, integrating functional and structural biology with genomic methods [4,5]. Collectively, the overall consensus of these studies permitted defining IRES elements by their intrinsic functional features. Specifically, IRES elements promote internal initiation of translation, independently of the 5′ cap structure. This key hallmark confers the capability to initiate translation even under strong repressive cap-dependent conditions. Therefore, IRES elements remain active during eIF4G proteolytic inactivation, as typically shown in picornavirus-infected cells [6].

Despite sharing functional properties, IRES elements are a diverse type of RNA regulatory regions, frequently present in non-coding regions of mRNAs, with different structural organizations and also dissimilar protein requirements [7–9]. Furthermore, IRES elements are autonomous entities, conferring internal initiation of translation outside of their natural genetic context, thereby retaining the potential to direct protein synthesis in eukaryotic vectors [10]. In recent years, ribosome profiling, RNA structure mapping, structural analyses of macromolecular complexes, and gene editing have provided key evidence for the understanding of temporal and spatial mRNA translation in several organisms. The diverse sequence and structure of RNA molecules conferring internal initiation supports the notion that these regulatory elements could have evolved through strong selective pressures, providing an ideal system for genetic and evolutionary studies [11]. Additionally, recombination events occurring during virus infection in nature generate IRES elements with unique properties that endorse novel tissue tropisms and/or host-range spectrum [12].

## Viral IRES elements: modular RNA structure

As previously mentioned, IRES elements were initially discovered in the RNA genome of picornaviruses. In contrast with cellular mRNAs, all picornavirus genomes, which consist of a positive single-stranded monocistronic RNA ranging between 7 to 10 kb, lack a cap structure at the 5′ end, although they are polyadenylated at the 3′ end [6]. Hence, translation initiation in picornavirus RNA depends exclusively on the IRES activity promoted by sequences located on the 5′ UTR, which facilitate RNA–protein interactions among the viral RNA and host proteins, enabling picornavirus RNA to hijack the host translation machinery for its own benefit.

Most viral IRES elements adopt specific RNA structures, which serve as the anchoring site for the ribosome guided by RNA–RNA and/or RNA–protein interactions [13,14]. Nevertheless, the IRES elements present in the genomes of different RNA virus families do not share overall conserved features and also differ in the factors needed to assemble initiation complexes [7]. Hence, viral IRES elements are classified in distinct types according to their mechanism of translation initiation. Translation of the enterovirus, cardiovirus, and aphthovirus RNAs is independent of eIF4E but requires the central region of eIF4G, which remains in the C-terminal cleaved products following Lb or 2A picornavirus proteases [15–17], together with eIF4A, eIF2, and eIF3 to assemble 48S initiation complexes *in vitro* [18,19]. Another group of picornaviruses uses IRES elements known as hepatitis C virus (HCV)-like because of the similarity with the IRES of the HCV, which depends on eIF2 and eIF3 but is independent of eIF4G [12,13,20]. The pestivirus and some flavivirus genomes also use HCV-like IRES elements to drive translation initiation [21,22].

Further increasing the diversity of RNA elements conferring internal initiation of translation, a unique type of IRES is present in the intergenic region (IGR) of dicistrovirus genomic RNA. The IGR IRES folds as a three-pseudoknot (PK) structure, in which PKs II and III (PKII and PKIII) enable ribosome binding, while PKI resembles a tRNA-like anticodon stem-loop, allowing the assembly of the initiation complex without eIFs (Figure 1B) [23]. Nevertheless, the diversity of IRES elements in viruses infecting invertebrates is astonishing [24,25], indicating that understanding the relationship between RNA structure and function remains a critical challenge in the RNA biology field.

Regarding picornavirus RNA conformation, the IRES elements of enterovirus, cardiovirus, and aphthovirus share a similar modular organization (Figure 1B). The enterovirus IRES comprises stem-loops 2–6, while cardio- and aphthovirus IRES elements are composed of stem-loops 2–5 (or H-L) [26,27]. In both cases, the central domain folds as a cruciform structure, comprising an internal C-rich loop and a GNRA (N stands for any nucleotide and R for purine) tetraloop, essential for IRES activity [28]. Interactions between nucleotides belonging to the GNRA tetraloop and the C-rich bulge promote long-range interactions, enabling the formation of a constrained structure that likely provides the correct orientation to recruit the ribosome subunits to the initiation site [29,30]. Furthermore, the A-pentaloop bulge of domain J-K acts as a docking site for base-pair receptors in concerted action of all subdomains [31], although a construct harboring domains J-K alone is not sufficient to promote IRES activity [32]. Similarly, the hepatitis A virus IRES contains a three-way junction structure, organized by an adenine-rich stem-loop motif [14]. Therefore, the topological conservation observed among these IRES elements suggests that a three-way junction behaves as a scaffold to pre-organize helical domains for recruiting the translation machinery.

Structural and functional analyses indicate that RNA flexibility is a prominent feature of picornavirus IRES elements. In particular, selective 2′-hydroxyl acylation analyzed by primer extension (SHAPE) and base-specific chemical probes revealed a realignment of major domains in enterovirus and aphthovirus IRES, including long-range interaction between specific RNA domains [33,34]. These structural features suggest a hierarchical folding process that couples RNA structure to IRES function during picornavirus infection. In line with this idea, sequence covariation in field virus isolates reveals evolutionarily constrained variability, which preserves the secondary structure of IRES elements critical for ribosome anchoring activity [27]. Beyond maintaining base pairing in stems, conserved bases in exposed loops and the junctions connecting stems highlight the relevance of these motifs for RNA function.

Additionally, SHAPE structure probing revealed that the IRES structure is flexible at near-physiological concentration of Mg^2+^ [35], indicating a dynamic conformation that allows the design of compounds inhibiting IRES-dependent protein synthesis under physiological conditions. Thus, the predominant role of RNA structure conformation for IRES activity allows the design of small molecules targeting specific conserved motifs to interfere with virus multiplication [36,37]. Consistent with this idea, incubation of the enterovirus IRES with dimethylamiloride-135 results in the allosteric stabilization of a ternary complex involved in the interaction with the AU-rich element/poly(U)-binding/degradation factor 1 (AUF1) protein, repressing translation initiation [38].

## RNA-binding protein involvement in IRES function

A significant number of diverse RNA-binding proteins (RBPs) regulate IRES function in various ways. Some of them stabilize the interaction of the IRES element with eIFs, mediating recruitment of the 40S ribosomal subunit, while others unwind RNA secondary structure near or at the start codon and/or titrate IRES ligands [39]. Notwithstanding, a different amount of proteins present in distinct cell types might contribute to cell type-dependent IRES function, likely restricting cross-kingdom IRES activity. Cumulative data indicate that RBPs interact with RNA targets responding in a co-ordinated manner to changes in the cell environment, as well as to intracellular signals. Therefore, understanding how and when combinations of these proteins bind to IRES elements to assemble a functional ribonucleoprotein (RNP) complex will bring new insights into the strategies used by RNA viruses to express their genome.

Conserved RNA motifs have been explored as candidates for RBP interaction with the aim to understand the mechanism promoting internal initiation. Over the years, different researchers have identified RBPs typically involved in nuclear and cytoplasmic RNA metabolism, associated with viral IRES elements. A few examples of factors stimulating IRES activity are ErbB3-binding protein 1, heterogeneous nuclear ribonucleoprotein K, DExD-box helicase 21, or ribosomal protein 13 via the Aspartic-Glutamic-Alanine-Aspartic (DEAD)-box helicase DDX3, among others [40–43]. Typical conserved motifs found in several IRES elements are the polypyrimidine tract, recognized by polypyrimidine binding protein, and the adenine-cytosine (ACCCC) sequence loop, recognized by poly(rC)-binding protein 2 [44]. These proteins assist eIFs in reconstitution assays by favoring a functional RNA structure conformation that stimulates IRES activity [45].

Nevertheless, not all RBPs interacting with IRES elements recognize conventional RNA-binding motifs, opening the question of whether this is due to unspecific binding. The relevance of these RBPs for IRES activity has been shown by functional studies in infected cells, as in the case of DEXD/H-box RNA helicase (DDX60), which negatively regulates translating ribosome activity on type II IRES-driven reporters and viral mRNA [46], or the Gem-associated protein 5 (Gemin5), whose IRES-dependent translation activity depends on its proteolytic cleavage by the foot-and-mouth disease leader b (Lb) protease (FMDV Lb protease) in infected cells [47,48]. Therefore, the role of IRES-interacting factors (ITAF) in IRES activity reflects the dynamic composition of RNP complexes in response to distinct signaling pathways, which modify the conformational activity of the IRES or assist in the interaction of the IRES with eIFs, mediating the recruitment of the ribosomal subunits.

Conserved RNA motifs have been explored as candidates for RBP interaction with the aim to understand the mechanism promoting internal initiation. Over the years, different researchers have identified RBPs typically involved in nuclear and cytoplasmic RNA metabolism, associated with viral IRES elements. A few examples of factors stimulating IRES activity are ErbB3-binding protein 1, heterogeneous nuclear ribonucleoprotein K, DExD-box helicase 21, or ribosomal protein 13 via the DEAD-box helicase DDX3, among others [40–43]. Typical conserved motifs found in several IRES elements are the polypyrimidine tract, recognized by polypyrimidine binding protein, and the ACCCC loop, recognized by poly(rC)-binding protein 2 [44]. These proteins assist eIFs in reconstitution assays by favoring a functional RNA structure conformation that stimulates IRES activity [45]. Nevertheless, not all RBPs interacting with IRES elements recognize conventional RNA-binding motifs, opening the question of whether this is due to unspecific binding. The relevance of these RBPs for IRES activity has been shown by functional studies in infected cells, as in the case of DEXD/H-box RNA helicase (DDX60), which negatively regulates translating ribosome activity on type II IRES-driven reporters and viral mRNA [46], or the Gem-associated protein 5 (Gemin5), whose IRES-dependent translation activity depends on its proteolytic cleavage by the FMDV Lb protease in infected cells [47,48]. Therefore, the role of ITAFs in IRES activity reflects the dynamic composition of RNP complexes in response to distinct signaling pathways, which modify the conformational activity of the IRES or assist in the interaction of the IRES with eIFs, mediating the recruitment of the ribosomal subunits.

## Cellular IRES elements

Historically, RNA-dependent processes initially discovered in viruses, such as splicing, polyadenylation, or cap-dependent translation, were subsequently found in cellular mRNAs [49–51]. Not surprisingly, a subset of RNA regions, tentatively termed cellular IRES elements, was reported to promote cap-independent translation under translation repressive scenarios [52–54]. However, these elements differ in many ways from well-documented viral IRES elements, including the primary requirements needed to trigger protein synthesis, raising controversies and doubts against cellular IRES elements, at least in part due to experimental design [55]. Despite the nomenclature given to these RNA elements, it cannot be ignored that several cellular mRNAs are translated in a cap-independent manner under cellular stress, as shown by the anti-apoptotic gene Bcl2 mRNA enrichment in polysomal RNA fractions [56]. Hence, the controversy raised about IRES activity validation has led to the implementation of novel methods. For instance, a circular RNA (circRNA) comprising a split nanoluciferase reporter has been proposed as a reliable method for screening IRES activity [57]. Indeed, circRNA reporters used to assess previously assigned and new IRES elements revealed that all viral IRES elements tested, but only some cellular IRES elements, exhibit internal initiation in circRNA reporters [58].

One of the key features of the putative cellular IRES elements is their presence in mRNAs that normally are translated in a cap-dependent manner, such that IRES-dependent translation generates alternative proteins, likely differing in function from the conventional cap-dependent protein synthesis of the same mRNA. For instance, IRES-dependent expression of Huntingtin-interacting protein K (HYPK) mRNA generates a truncated protein, HYPK-ΔN, lacking the tri-arginine motif that acts as the nuclear localization signal [59]. Therefore, while the full-length HYPK protein translocates to the nucleus preventing the aggregation of a mutant p53 protein, the HYPK-ΔN lacks this activity, compromising the ability of HYPK protein to regulate the cell cycle.

As mentioned above, comparison of sequence and secondary structure of cellular IRES elements shows no overall similarities. The 5′ end of the chaperone heat shock protein 70 mRNA forms a compact structure with multiple stable stems, different from picornavirus IRES elements, that regulates translation in a cap-independent manner [60]. However, the structural analysis of a stem-loop within the Jun Proto-Oncogene (c-JUN) 5′ UTR shows similarity to the eIF3 binding motifs found in HCV-like IRES elements, suggesting partial mechanistic similarities [61]. In other cases, the putative IRES region includes G-rich sequences predicted to fold as guanine quadruplex [62,63].

Beyond the variability in RNA structure, there are also differences in eIFs recruitment in cellular mRNAs, which undergo cap-independent translation. Similar to some viral RNAs, the IRES elements driving translation of the murine insulin receptor and the insulin-like growth factor 1 receptor transcripts are resistant to eIF4F inhibition but require the highly charged and disordered N-terminus of eIF5B [64]. In other cases, eIF4A and eIF4E enhanced eIF4GI binding affinity to the uncapped 5′ UTR of hypoxia-inducible factor 1 subunit alpha mRNA, whose translation initiation is driven by a 3′ cap-independent translation element. In contrast, eIF4A and eIF4E have no effect on the binding of eIF4GI to the 5′ UTR of fibroblast growth factor 9 mRNA [65].

In addition to eIFs, there are cellular IRES elements activated by ubiquitous RBPs, such as splicing factor proline and glutamine rich (SFPQ) or heterogeneous nuclear ribonucleoprotein (hnRNP) A1. The 5′ UTR of casein kinase 1α (CK1α) mRNA functions as a putative IRES element in human carcinoma colorectal cancer cell lines (HCT)-116 colon cancer cells, such that silencing of SFPQ reduced CK1α protein abundance and partially blocked RAS-mutant colon cancer cell growth [66]. In other cases, translation of cellular IRES reporters (cyclin D1 and c-Myc) has been shown to be dependent on methylation of hnRNP A1 by the type II arginine methyltransferase protein arginine methyltransferase (PRMT) [67]. Notably, IRES activity is not uniquely found in animal cells. In plants, defense mRNAs with a purine-rich (R) motif are selectively translated via eIFiso4G over the repressive eIF4G. Specifically, R-motif-dependent translation is driven by PABPs through association with the pattern-triggered immunity (PTI), which induces mRNA decapping. Phosphorylation by PTI regulators mitogen-activated protein kinase 3 and 6 inhibits eIF4G’s activity while enhancing PABP binding to the R-motif and promoting eIFiso4G-mediated defense mRNA translation [68].

The recent development of novel computational methods allowed the rapid prediction of putative IRES elements in mRNAs, long non-coding RNAs, and circRNAs (Figure 1C) [69,70], which, however, need to be validated in functional assays. CircRNAs are covalently closed, mostly cytosolic RNAs present in eukaryotes. Despite the lack of a free 5′ end, some of these RNAs are translated, generating microproteins that are shorter than the mRNA-encoded proteins [71,72]. IRES-dependent translation of circRNAs has been proposed to be mediated by short sequences complementary to 18S rRNA [73]. In other cases, N6-methyladenosine (m(6)A) RNA modification, adenosine to inosine RNA editing, and the interaction with the eIF4A3 component of the exon junction complex have been proposed to drive cap-independent initiation [74]. Translation of several circRNAs has been verified in a cell-based system [75], wherein overrepresented hexamers appear to undergo cap-independent initiation of translation, generating low-abundance short small open reading frames (smORFs)-coded peptides, although sufficient to be identified by mass spectrometry.

## Impact of RNA methylation in IRES activity and cap-independent translation

RNA modifications not only regulate RNA stability but also translation efficiency. The impact of RNA modifications, known as the epitranscriptome, on IRES-dependent activity has been reported in cells with ribosomal RNA (rRNA) 2′-O methylation (2′-O-Me) and also in cases with mRNA m(6)A modification. In eukaryotic cells, ribose 2′-O-Me, the most abundant rRNA chemical modification, is mediated by the rRNA methyl-transferase fibrillarin (FBL) [76]. The intrinsic capability of ribosomes to translate mRNAs depends upon the 2′-O-Me pattern, as shown with reporters using the dicistrovirus IGR IRES. Furthermore, the enhancer of zeste homologue 2 (EZH2), a lysine methyltransferase, interacts with FBL regulating translational regulation. In fact, EZH2 deficiency impairs global translation and reduces IRES-dependent translation initiation in cancer cells [77].

The m(6)A-modified base is the most abundant RNA post-transcriptional modification, known to be present in non-coding RNAs, ribosomal RNAs, polyadenylated RNAs, and mRNAs. Initially, the inactivation of the fat mass and obesity-associated gene (Fto), encoding a nucleic acid demethylase that impairs dopamine receptor-dependent control of neuronal activity and behavioral responses, demonstrated the relevance of RNA methylation for gene expression regulation [78]. Subsequently, the prevalence of this modification was redefined by transcriptome-wide m(6)A mapping studies [79]. In mammalian cells, the asymmetric distribution of m(6)A along mRNAs results in diminished methylation in the 5′ UTR compared with other regions. However, certain adenosines within the 5′ UTR of newly transcribed mRNAs are preferentially methylated in response to heat shock stress. In this situation, the nuclear m(6)A reader YT521-B homology domain (YTH) m6A RNA-binding protein 2 (YTHDF2) preserves 5′ UTR methylation of stress-induced transcripts by limiting the m(6)A eraser FTO from demethylation, and as a consequence, the increased 5′ UTR m(6)A methylation promotes cap-independent translation initiation, providing a mechanism for selective mRNA translation under heat shock stress [80].

The identification of adenosine methyltransferases (writers), m(6)A demethylating enzymes (erasers), and m(6)A-binding proteins (readers) defined novel cellular pathways for the post-transcriptional regulation of mRNAs. The work by Meyer et al. [81] showed that the presence of 5′ UTR m(6)A promotes eIF3 binding, recruiting the 43S complex to initiate translation in the absence of eIF4E, while inhibition of adenosine methylation selectively reduces translation of mRNAs containing 5′ UTR m(6)A. Importantly, m(6)A facilitates mRNA translation that is resistant to eIF4F inactivation [82] but requires an accessible 5′ terminal end on the mRNA, indicating that m(6)A-containing 5′ UTRs are distinct from viral IRES elements [82]. Curiously, depletion of the methyltransferase METTL3 selectively inhibits translation of mRNAs bearing 5′ UTR methylation, but not mRNAs with 5′ terminal oligopyrimidine elements, which require a distinct subset of proteins to initiate translation. More recently, the role of m(6)A was investigated for HCV IRES-dependent translation, mutating m(6)A consensus motifs (DRACH) located upstream of the initiator codon. Interestingly, the m(6)A protein YTH N6-methyladenosine RNA-binding protein C2 (YTHDC2), which contains the RNA helicase domain, recognizes m^6^A-methylated RNA supporting IRES-dependent translation [83].

## Conclusions

The function and significance of a wide variety of viral IRES elements, and particularly putative IRES elements predicted in RNAs still remain poorly understood, particularly when these elements drive synthesis of smORFs or microproteins, currently hard to verify and validate the functional relevance of the corresponding polypeptide. The wide diversity of translation initiation mechanisms that evolved in viruses and various living organisms is currently under exhaustive exploration using genomics, metagenomics, epitranscriptomics, and in some cases by proteomics. However, lessons learned from earlier studies of putative IRES elements highlight the key challenges and progress that has been made within the RNA translation field in the last decades. Therefore, the IRES elements predicted by computational methods require a deep stringent functional analysis, as recommended by researchers working in the field [84].

**Perspectives**

- Internal ribosome entry site (IRES) elements, initially found in picornavirus RNAs, mediate internal cap-independent translation initiation through a modular RNA structure organization, which serves as the anchoring site for the ribosome often guided by various RNA–protein interactions.
- Translation initiation mechanisms operating in eukaryotic organisms are under exhaustive exploration using genomics, and particularly computational biology, predicting putative IRES elements conferring cap-independent translation that need to be functionally validated.
- Lessons learned from functional and structural studies of IRES elements found in RNA viruses, and also in cellular mRNAs, highlight key challenges, controversies, and progress made in the minimal requirements needed in the design and interpretation of cap-independent data.

## Abbreviations

ACCCC: adenine-cytosine (ACCCC) sequence
CK1α: casein kinase 1α
DEAD: Aspartic-Glutamic-Alanine-Aspartic
EZH2: zeste homologue 2
FBL: fibrillarin
FMDV Lb protease: Foot-and-mouth disease leader b (Lb) protease
HCT: human carcinoma colorectal cancer cell lines
HCV: hepatitis C virus
HYPK: Huntingtin interacting protein K
IGR: intergenic region
IRES: internal ribosome entry site
ITAF: IRES-interacting factor
M^7^GTP: methyl-7-GTP (cap)
2′-O-Me: 2′-O methylation
ORFs: open reading frames
PABP: poly(A)-binding protein
PK: pseudoknot
PRMT: protein arginine methyltransferase
PTI: pattern-triggered immunity
RBP: RNA-binding protein
RNP: ribonucleoprotein
SFPQ: splicing factor proline and glutamine rich
SHAPE: selective 2′-hydroxyl acylation analyzed by primer extension
UTR: untranslated region.
YTH: YT521-B homology domain
circRNA: circular RNA
eIFs: eukaryotic initiation factors
hnRNP: heterogeneous nuclear ribonucleoprotein
m(6)A: N6-methyladenosine RNA modification
rRNA: ribosomal RNA
smORFs: small open reading frames
